# Regulation of the Peptidoglycan Polymerase Activity of PBP1b by Antagonist Actions of the Core Divisome Proteins FtsBLQ and FtsN

**DOI:** 10.1128/mBio.01912-18

**Published:** 2019-01-08

**Authors:** Adrien Boes, Samir Olatunji, Eefjan Breukink, Mohammed Terrak

**Affiliations:** a InBioS-Centre d’Ingénierie des Protéines, Liège University, Liège, Belgium; b Membrane Biochemistry and Biophysics, Department of Chemistry, Faculty of Science, Utrecht University, Utrecht, The Netherlands; University of Amsterdam; Fred Hutchinson Cancer Research Center

**Keywords:** divisome, FtsBLQ, FtsN, lipid II, PBP1b, peptidoglycan

## Abstract

Peptidoglycan (PG) is an essential constituent of the bacterial cell wall. During cell division, PG synthesis localizes at midcell under the control of a multiprotein complex, the divisome, allowing the safe formation of two new cell poles and separation of daughter cells. Genetic studies in Escherichia coli pointed out that FtsBLQ and FtsN participate in the regulation of septal PG (sPG) synthesis; however, the underlying molecular mechanisms remained largely unknown. Here we show that FtsBLQ subcomplex directly interacts with the PG synthase PBP1b and with the subcomplex FtsW-PBP3, mainly via FtsW. Strikingly, we discovered that FtsBLQ inhibits the glycosyltransferase activity of PBP1b and that this inhibition was antagonized by the PBP1b activators FtsN and LpoB. The same results were obtained in the presence of FtsW-PBP3. Moreover, using a simple thioester substrate (S2d), we showed that FtsBLQ also inhibits the transpeptidase domain of PBP3 but not of PBP1b. As the glycosyltransferase and transpeptidase activities of PBP1b are coupled and PBP3 activity requires nascent PG substrate, the results suggest that PBP1b inhibition by FtsBLQ will block sPG synthesis by these enzymes, thus maintaining cell division as repressed until the maturation of the divisome is signaled by the accumulation of FtsN, which triggers sPG synthesis and the initiation of cell constriction. These results confirm that PBP1b plays an important role in E. coli cell division and shed light on the specific role of FtsN, which seems to counterbalance the inhibitory effect of FtsBLQ to restore PBP1b activity.

## Abstract (precis)

Bacterial cell division is governed by a multiprotein complex called divisome, which facilitates a precise cell wall synthesis at midcell and daughter cell separation. Protein-protein interactions and activity studies using different combinations of the septum synthesis core of the divisome revealed that the glycosyltransferase activity of PBP1b is repressed by FtsBLQ and that the presence of FtsN or LpoB suppresses this inhibition. Moreover, FtsBLQ also inhibits the PBP3 activity on a thioester substrate. These results provide enzymatic evidence of the regulation of the peptidoglycan synthase PBP1b and PBP3 within the divisome. The results confirm that PBP1b plays an important role in E. coli cell division and shed light on the specific role of FtsN, which functions to relieve the repression on PBP1b by FtsBLQ and to initiate septal peptidoglycan synthesis.

## INTRODUCTION

Peptidoglycan (PG) is the major constituent of the bacterial cell wall; it surrounds the cytoplasmic membrane, determines the cell shape, and protects the cell from rupture under its internal osmotic pressure. PG polymerization starts with the lipid II precursor, which is synthesized on the inner face of the cytoplasmic membrane and subsequently needs to be translocated through this membrane by the MurJ flippase (1). FtsW of the SEDS (shape, elongation, division, and sporulation) protein family was also proposed previously as a lipid II flippase (2), and a new study proposes that both FtsW and MurJ are required for lipid II transport (3). Once on the periplasmic side of the membrane, lipid II is polymerized by the glycosyltransferase (GTase) activities of the class A PBPs (aPBPs) and SEDS proteins and the transpeptidase (TPase) activities of aPBPs and class B PBPs (bPBPs) (4–8). Inhibition of either aPBPs or rod system (SEDS) reduces PG synthesis by about 80%, indicating that both synthase systems are required for optimal cell wall assembly and may collaborate (9).

For the safe growth and division of the bacterial cell, PG synthesis and also hydrolysis proceed without compromising the integrity of the cell, thanks to the precise coordination and the tight regulation of the enzymes within multiprotein complexes, the elongasome and the divisome, respectively, active in cell elongation and division (10–12). The divisome governs septal PG (sPG) synthesis, cell envelope constriction, and cell separation at midcell (13, 14). In Escherichia coli, this dynamic machinery includes more than 20 essential and accessory proteins which assemble in an ordered and interdependent manner in two steps (15); first, the tubulin-like FtsZ, ZipA, FtsA, ZapA to -E, and FtsEX localize at midcell in contact with the inner face of the cytoplasmic membrane. Later, the downstream components FtsK, FtsB-FtsL-FtsQ, FtsW-FtsI (PBP3), and FtsN join sequentially, and FtsQLB and FtsW-PBP3 localize as preformed subcomplexes (16, 17), to complete the assembly of the mature divisome (18, 19). In addition, other proteins such as the PG synthase PBP1b and proteins that regulate its activities (LpoB, CpoB, and TolA) also associate with the divisome (13, 20). PBP1b (aPBP) is a bifunctional GTase/TPase enzyme responsible for both glycan chain polymerization and peptide cross-linking of the PG, respectively (21, 22). Its activity is stimulated by FtsN and the outer membrane (OM) lipoprotein LpoB; the latter binds to the UB2H domain of PBP1b (23–26). In E. coli, PBP1b requires FtsW and PBP3 for septal localization (13, 20), and the three proteins were shown to form a ternary complex (27), the septal synthase subcomplex of the divisome. Several studies have shown interactions between the components of this subcomplex and FtsN and FtsBLQ subunits (10, 17, 26, 28–32). FtsW is a polytopic membrane protein that plays an essential role in cell division. We have shown that FtsW directly interacts with lipid II and that PBP3 regulates this interaction and the availability of the substrate for the PBP1b synthase (27), but the GTase activity was not detected with three different FtsW proteins or the E. coli FtsW-PBP3 complex (27). Recently, FtsWs from different bacteria have been shown to polymerize lipid II into PG in a bPBP-dependent manner (6). PBP3 (bPBP) is a monofunctional TPase specific and essential for septal PG synthesis and cell constriction (33, 34); its activity requires prior glycan chain elongation by a GTase. Streptococcus thermophilus PBP2x was able to cross-link glycan chain provided by its cognate FtsW and noncognate aPBP (whose TPase was inactivated) and a monofunctional GTase (6), suggesting that E. coli PBP3 may also use the glycan substrate formed by PBP1b and FtsW.

Homologues of FtsBLQ are genetically well conserved among bacterial species and are absent from bacteria without a cell wall (35, 36). In E. coli, soluble constructs of FtsBLQ form a ternary complex in a 1:1:1 stoichiometry (37); however, the membrane forms of FtsBL were shown to assemble as an L_2_B_2_ tetramer (38). FtsB, FtsL, and FtsQ are bitopic membrane proteins of 103, 121, and 276 residues, respectively, composed of a short N-terminal cytoplasmic sequence, a transmembrane segment, and a periplasmic domain (35, 39). The latter is divided into two subdomains in FtsQ, the α- and β-domains. The α-domain with a polypeptide transport-associated (POTRA) fold was shown to be involved in transient protein-protein interactions (40). The β-domain interacts with many divisome proteins, including FtsB/FtsL. The last C-terminal 30 amino acids are not structured and play an important role in the interaction with FtsB/L. FtsBLQ are essential proteins of the divisome; they were long believed to have a scaffolding function (35, 39), and only recently an active role in the regulation of septal PG synthesis was attributed to this protein complex in coordination with FtsA and FtsN (41, 42). Mutations in FtsB or FtsL lead to premature initiation of constriction, and the mutants completely or partially bypass the need for FtsN, FtsK, ZipA, and FtsA.

FtsN is a bitopic membrane protein composed of a small cytoplasmic domain that interacts with FtsA at the 1C subdomain, a transmembrane α-helix, and a large periplasmic domain (43, 44). The latter is divided into three subdomains, including a membrane-proximal portion containing three short α-helices; the region located around α-helix 2 (L75 to Q94, ^E^FtsN) is essential for the function of the protein (41, 45), and it is followed by a glutamine-rich central region and a PG binding SPOR domain at the C terminus which binds preferentially to glycan chains devoid of stem peptides (46–48). FtsN is the last essential protein to localize at the division site; its accumulation using a positive feedback mechanism completes the maturation of the divisome and triggers the initiation of cell envelope constriction (45).

In this work, we have investigated the role of the complex FtsBLQ in peptidoglycan synthesis *in vitro* and the coordination of its activity with that of FtsN. We first analyzed the interactions of FtsBLQ and FtsN with each other and with the synthase subcomplex components PBP1b-FtsW-PBP3. We then studied the effect of the observed interactions on the synthase activity of PBP1b with the lipid II substrate. Remarkably, we found that the complex FtsBLQ inhibits the GTase activity of PBP1b alone or in the presence of FtsW-PBP3 and that this inhibition is antagonized by FtsN or LpoB to restore PBP1b activity. Interestingly, using a thioester substrate, we showed that FtsBLQ also inhibits PBP3 activity. These results provide enzymatic evidence of the regulation of the PG synthase PBP1b and PBP3 within the divisome.

## RESULTS

### Expression and purification of the FtsBLQ complex.

**FIG 1:**
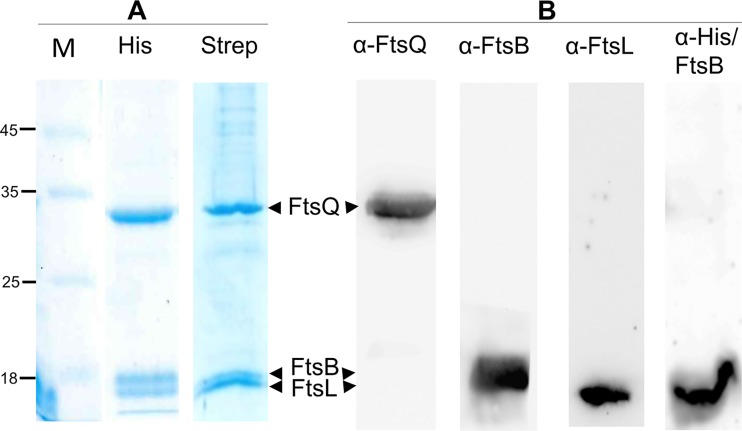
Purification of the FtsBLQ ternary complex and Western blot analysis. (A) SDS-PAGE analysis of HisFtsBLQ and StepFtsBLQ complexes purified on HisTrap (His) or StrepTrap (Strep), respectively. Numbers at left are molecular masses in kilodaltons. (B) Western blot analyses performed on HisFtsBLQ complex purified on HisTrap using specific antibodies against each protein and anti-His antibody for His-FtsB.

The full-length membrane forms of FtsB, FtsL, and FtsQ were coexpressed from one Duet plasmid in E. coli membranes, solubilized with DDM detergent, and copurified by affinity chromatography using a His tag (His) or a Strep tag at the N terminus of FtsB; the untagged FtsL and FtsQ coeluted with tagged FtsB, indicating the formation of a stable trimeric complex as expected from previous studies (Fig. 1A) (16). The presence and the identity of the three proteins in the purified complex were verified using specific antibodies against each one of them and anti-His antibodies for His-FtsB. The results show a single band with the expected molecular mass corresponding to His-FtsB, FtsL, and FtsQ (Fig. 1B).

**FIG 2:**
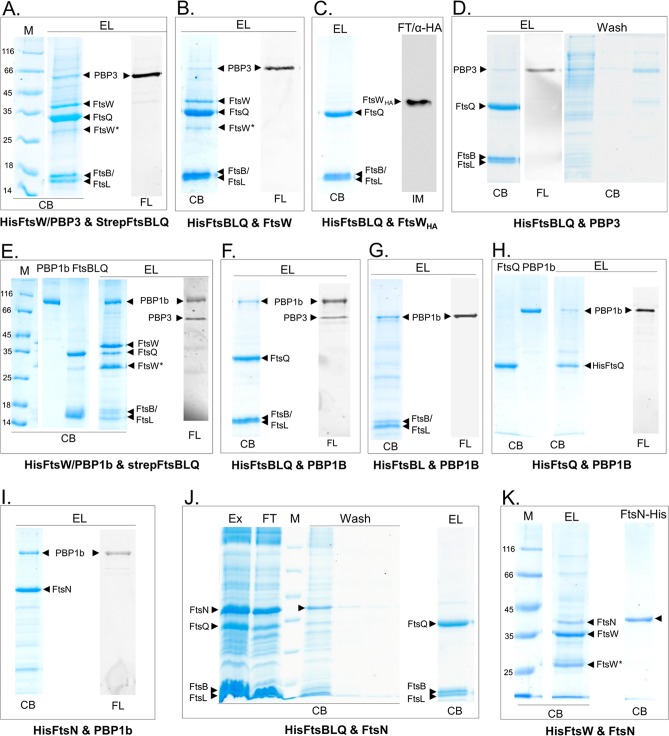
Protein-protein interactions using coexpression and copurification. The proteins indicated below each panel (A to K) were coexpressed in E. coli and copurified on a nickel column using one (His)-tagged protein as a bait. The eluted proteins were then labeled with fluorescent ampicillin, when PBPs were present, and analyzed by SDS-PAGE followed by fluorescence (FL) imaging and protein staining with Coomassie blue (CB). M, protein standard; EL, elution fractions from Ni affinity column; FL, fluorescently labeled PBPs; Ex, protein extracts; FT, indicates the flowthrough fractions. The bands of the proteins are indicated by arrowheads. FtsW* is a degradation product of FtsW. α-HA indicates immunoblotting (IM) analysis using antibodies against the HA epitope of FtsW_HA_. Numbers at left of panels are molecular masses in kilodaltons.

We then set out to study the interaction between FtsBLQ, FtsW and/or PBP3, PBP1b, and FtsN (only one protein that contained a His or Strep tag was used as a bait) by performing several coexpressions from compatible Duet vectors in E. coli, followed by the extraction of multiprotein complexes from the bacterial membranes using detergent and affinity copurification on a HisTrap or StrepTrap column. The proteins recovered were then labeled with fluorescent ampicillin, when PBPs were present, and analyzed by SDS-PAGE followed by fluorescence imaging and protein staining (Fig. 2). In all cases, we confirmed that the preys (nontagged or containing a different tag not compatible with the column used) do not bind to the column matrix.

### FtsBLQ interacts with FtsW/PBP3 and PBP1b but not with FtsN.

In the first coexpression experiment, we used HisFtsW/PBP3 and StrepFtsBLQ constructs (Strep tag on FtsB and His on FtsW and vice versa); the copurification results on a Ni column in both cases showed that FtsW/PBP3 and FtsBLQ coeluted, indicating an interaction between the two subcomplexes (Fig. 2A and data not shown). The quality of the purification using Strep tag was less efficient (most of the Strep-tagged protein was found in the flowthrough), and we used only His tag and the Ni-affinity column in subsequent experiments. When HisFtsBLQ was coexpressed with untagged FtsW or PBP3 separately, FtsW was found in the elution fraction (Fig. 2B), but most of PBP3 was recovered in the washing step, and only a small amount of PBP3 was retained by FtsBLQ (Fig. 2D). A larger amount of PBP3 coeluted with FtsBLQ when FtsW was also present, and even if PBP3 was not overexpressed, the endogenous PBP3 was copurified with FtsBLQ-FtsW (Fig. 2B). These results show that FtsBLQ directly interacts with FtsW with higher affinity than with PBP3, suggesting that the interaction of PBP3 with FtsBLQ is mainly mediated by FtsW. The results also indicate that the binding sites of FtsBLQ and PBP3 on FtsW are distinct. In contrast to FtsW, the mutant FtsW_HA_ (FtsW with the HA epitope inserted in the periplasmic loop between TM helices 7 and 8) was not coeluted with FtsBLQ (Fig. 2C), showing that the interaction between FtsBLQ and FtsW wild type is specific and that the large FtsW loop 7/8 plays a role in the binding.

By coexpressing HisFtsBLQ and PBP1b, we found that the untagged PBP1b (and endogenous PBP3) coeluted with HisFtsBLQ, showing that PBP1b interacts with the FtsBLQ complex (Fig. 2F). Similarly, the coexpression of HisFtsW/PBP1b and StrepFtsBLQ allowed the isolation of a six-protein complex which included the endogenous PBP3 (Fig. 2E), indicating simultaneous interaction between these proteins and that their binding sites are not overlapping. On the other hand, when the subunit HisFtsBL or HisFtsQ was coexpressed with PBP1b, the complexes FtsBL-PBP1b and FtsQ-PBP1b were formed, respectively, but a larger amount of PBP1b was coeluted with FtsBL than with FtsQ, suggesting that these FtsBLQ subunits interact directly with PBP1b, and the binding with FtsBL seemed higher than with FtsQ (Fig. 2G and H). Interestingly, while PBP3 coeluted with FtsBLQ-PBP1b, it was not present with FtsBL-PBP1b or FtsQ-PBP1b. This result excludes the possibility of PBP3 playing an intermediary role in the interaction between FtsBLQ and PBP1b.

FtsN was shown to interact with PBP1b and to stimulate its activity (26), and it was also suggested that FtsN regulates septal synthesis via FtsBLQ (41). Here we have confirmed the interaction between FtsN and PBP1b (Fig. 2I), but only a negligible amount of FtsN was copurified with HisFtsBLQ, and the bulk of the protein was present in the flowthrough and wash fractions (Fig. 2J). In contrast, a significant amount of FtsN was retained by HisFtsW (Fig. 2K). These results suggest that FtsN does not interact directly with or binds only weakly to the FtsBLQ subcomplex as observed previously (28, 31), but both FtsN and FtsBLQ seem to interact independently with the synthase subcomplex components FtsW-PBP3-PBP1b, confirming the presence of a large and dynamic divisome assembly. We then addressed the question of the significance of these interactions for the PBP synthase activities and regulation.

### Antagonist actions of FtsBLQ and FtsN on PBP1b activity.

**FIG 3:**
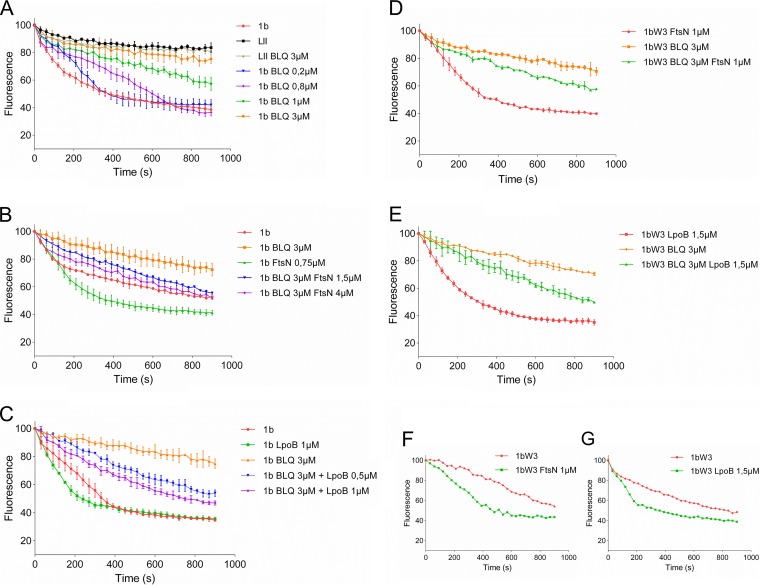
Effect of FtsBLQ and/or FtsN and LpoB on the activity of PBP1b in the presence and absence of FtsW/PBP3. The GTase activity of PBP1b with dansyl-lipid II is measured using a continuous fluorescence assay (the concentrations of PBP1b and other proteins and detergent concentrations were optimized for each experiment). Upon PG polymerization, the fluorescence decreases over time. Values are the mean ± SD from three experiments or a representative of three assays. (A) Inhibition of lipid II (LII) polymerization by PBP1b (1b) using variable concentrations of FtsBLQ (BLQ) subcomplex. (B and C) FtsN or LpoB, respectively, suppresses the inhibitory effect of FtsBLQ on PBP1b. (D and E) FtsN or LpoB, respectively, suppresses the inhibitory effect of FtsBLQ on PBP1b in the presence of FtsW/PBP3 (1bW3). (F and G) Activation of PBP1b by FtsN or LpoB, respectively, in the presence of FtsW/PBP3.

**FIG 4:**
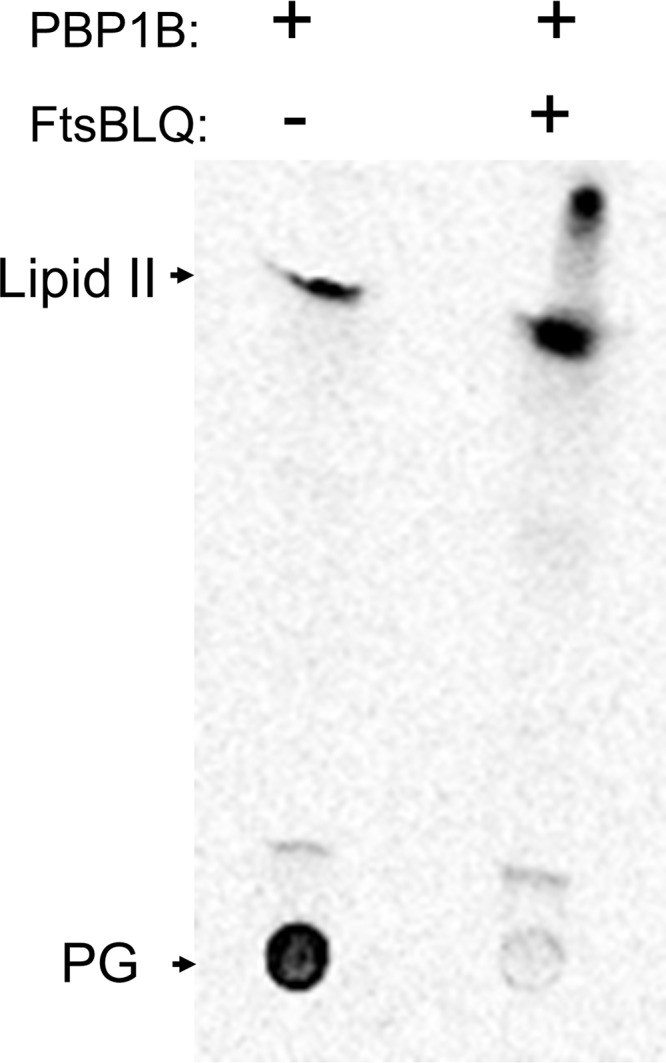
Inhibition of PBP1b GTase activity by FtsBLQ. TLC analysis of the reaction products formed by PBP1b from radioactive lipid II substrate. The addition of FtsBLQ in the PBP1b reaction mixture inhibits the polymerization of lipid II into PG polymer.

It was suggested that both FtsBLQ and FtsN participate in the regulation of septal peptidoglycan synthesis and the initiation of cell constriction (41), but the specific target of FtsBLQ and the molecular mechanisms were not identified. FtsN is known to interact with PBP1b (confirmed above) and to stimulate its activity, and we showed above that FtsBLQ also interacts with PBP1b. To understand the effects of these interactions on the enzymatic activity of PBP1b, we monitored its polymerase reaction by continuous fluorescence assay in the presence of FtsBLQ and/or FtsN using dansyl-lipid II as the substrate (49). This was first tested using the well-known PBP1b activators FtsN and LpoB. As expected, these two proteins stimulated the activity of PBP1b (Fig. 3B and C). We then tested the effect of the purified FtsBLQ subcomplex on the activity of PBP1b. Strikingly, we discovered that the addition of FtsBLQ inhibits the GTase activity of PBP1b in a concentration-dependent manner (Fig. 3A). This effect was confirmed using a second assay with radiolabeled lipid II and visualization of the reaction products (Fig. 4). This finding reveals for the first time the role of FtsBLQ as a negative regulator of the PBP1b synthase. Interestingly, we also found that the inhibitory effect of FtsBLQ on the activity of PBP1b can be relieved in the presence of FtsN (Fig. 3B) or LpoB (Fig. 3C). As PBP1b interacts with FtsW/PBP3, the same experiments were then performed in the presence of FtsW/PBP3 to test the combined effect of the interacting subcomplexes. Here also, we found that FtsBLQ represses the polymerase activity of PBP1b and that FtsN or LpoB allows its recovery in this environment, mimicking the enzymatic core of the divisome (Fig. 3D and E). The activation of PBP1b by FtsN or LpoB in the presence of FtsW/PBP3 was equally maintained (Fig. 3F and G). These results are well in line with the published *in vivo* data showing that for E. coli to survive in the absence of FtsN, suppressor mutations in FtsB or FtsL were also required (41). We conclude that the subcomplex FtsBLQ interacts with the sPG synthase subcomplex PBP1b-FtsW-PBP3 and maintains the activity of PBP1b as repressed until the arrival of FtsN, which triggers sPG synthesis and constriction of the bacterial cell.

### The FtsBL subunit inhibits PBP1b activity.

**FIG 5:**
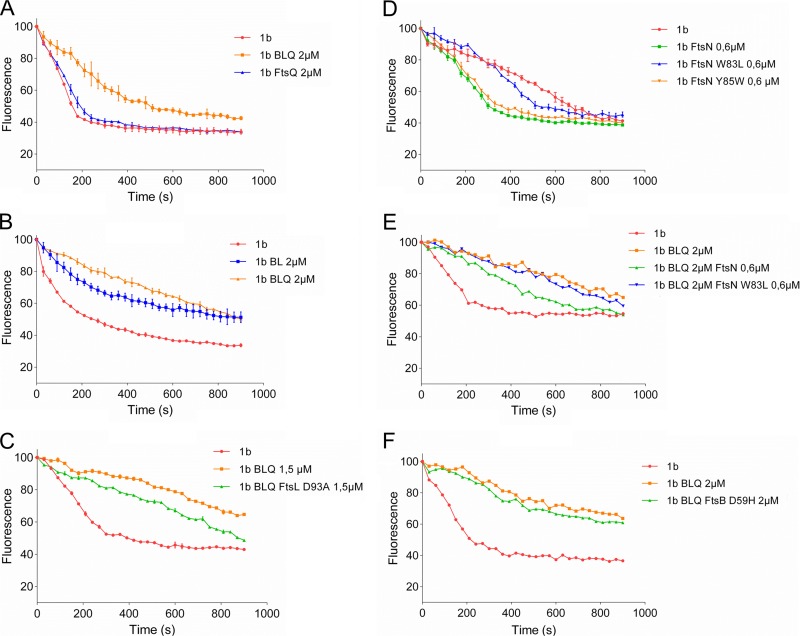
Effect of FtsBLQ subunits and mutants and FtsN mutants on the activity of PBP1b. The GTase activity of PBP1b was measured by continuous fluorescence assay (the concentration of PBP1b and other proteins and detergent concentration were optimized for each experiment). (A and B) Comparison of the effects of FtsBLQ with the subunit FtsQ or FtsBL, respectively, on the activity of PBP1b. (C) Comparison of the effect of FtsBLQ containing the mutation FtsL^D93A^ and control complex on the activity of PBP1b. (D) Comparison of the effects of the FtsN^W83L^ and FtsN^Y85W^ mutants and wild-type FtsN on the activity of PBP1b. (E) Comparison of the effects of FtsN and FtsN^W83L^ mutant on PBP1b inhibited by FtsBLQ. (F) Effect of FtsBLQ containing the mutation FtsB^D59H^ on the activity of PBP1b.

To better understand the mechanism of PBP1b inhibition by the FtsBLQ complex, subunits have been purified and tested. As FtsB and FtsL have been shown to be dependent on each other for stability, proper cellular localization, and function (39, 50), we compared the effect of the ternary subcomplex with that of the FtsBL dimer and FtsQ, which have been shown earlier to interact with PBP1b. We found that while FtsQ has no effect on the polymerase activity of PBP1b (Fig. 5A), the FtsBL dimer, in contrast, without detectable FtsQ by immunoblotting (see Fig. S1 in the supplemental material), exhibited high inhibition activity approaching that of the FtsBLQ subcomplex (Fig. 5B). These results indicate that FtsBL represents the main inhibitory factor of PBP1b while FtsQ is not directly involved in the inhibition. This is consistent with the finding that suppressor mutations that bypass FtsN were located in FtsB and FtsL and not in FtsQ (41).

10.1128/mBio.01912-18.2 FIG S1 Purification of FtsBL and immunoblotting using antibodies against FtsQ. Left, Coomassie blue-stained SDS-PAGE gel showing FtsB and FtsL bands indicated by arrows, M, molecular standards. Right (α-FtsQ), immunoblotting results confirming the absence of FtsQ in the purified FtsBL sample and FtsQ as control. Download FIG S1, PDF file, 0.3 MB. Copyright © 2019 Boes et al. 2019 Boes et al. This content is distributed under the terms of the Creative Commons Attribution 4.0 International license.

### The mutation FtsL^D93A^ decreases the inhibition of PBP1b by FtsBL^D93A^Q subcomplex.

**FIG 6:**
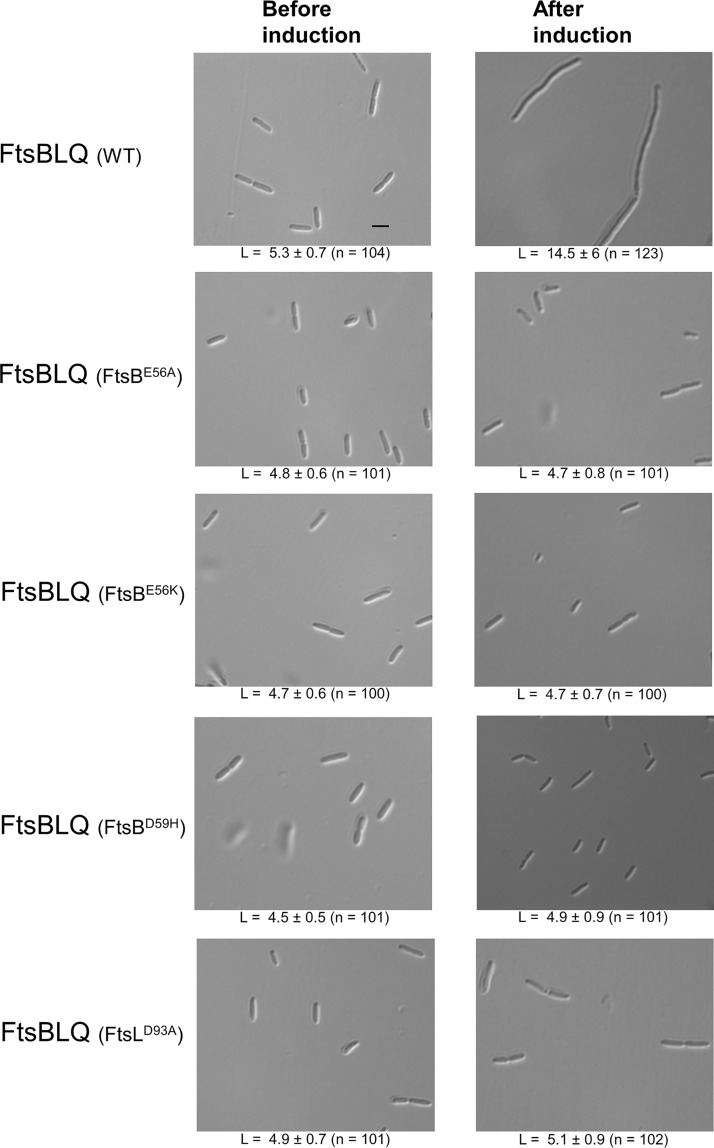
Phenotype of E. coli cells (C43 DE3) overexpressing FtsBLQ complex or mutants. FtsBLQ (WT) induces extensive cell filamentation after induction, while the cells overproducing FtsBLQ complexes with a mutation in FtsB or FtsL exhibit comparable cell length before and after induction. The average cell length (L) ± SD (µm) and the number of cells measured (n) are shown below the corresponding representative image. The average cell width in all cases was ∼1.3 µm. Bar, 5 μm.

FtsN-suppressor mutations located in the so-called constriction control domains (CCDs) of FtsB (A55 to D59) and FtsL (E88 to H94) have been shown to restore bacterial division in the absence of FtsN (41). This suggests that FtsBLQ complexes harboring these mutations are not functional, and in light of our results, we hypothesized that they might not inhibit PBP1b and thus no reactivation by FtsN would be needed. To test this hypothesis, FtsBLQ subcomplexes containing the single mutation FtsB^E56A^, FtsB^E56K^, FtsB^D59H^, or FtsL^D93A^ were purified and tested for their effect on PBP1b. All purified subcomplex variants were undistinguishable from that without these mutations in terms of expression level and complex recovery, indicating that these mutations did not affect the stability or dissociate the subcomplex (Fig. S2). Interestingly, we found that the inhibitory effect of the FtsBL^D93A^Q mutant decreased significantly compared to the control reaction (Fig. 5C). In contrast, the effects of FtsBLQ subcomplexes harboring FtsB^E56A^, FtsB^E56K^, or FtsB^D59H^ on the activity of PBP1b were comparable to those of FtsBLQ without these mutations (Fig. 5F and Fig. S3). These results suggest that FtsL is the specific inhibitor of the PBP1b GTase activity, with residue D93 playing an essential role in this process, and that FtsB plays a distinct role as proposed previously (41). Interestingly, induction of the expression of FtsBLQ results in long filaments, indicating an inhibition of cell division, while the cells producing FtsBLQ containing the FtsB^E56A^, FtsB^E56K^, FtsB^D59H^, or FtsL^D93A^ mutations exhibit cell length and width comparable to those before induction (Fig. 6), suggesting that these mutants are functionally inactive *in vivo*.

10.1128/mBio.01912-18.3 FIG S2 Coomassie blue-stained SDS-PAGE showing purified FtsBLQ complex and variants containing FtsB mutation E56A, E56K, or D59H or FtsL D93A. M, protein standard. Download FIG S2, PDF file, 0.2 MB. Copyright © 2019 Boes et al. 2019 Boes et al. This content is distributed under the terms of the Creative Commons Attribution 4.0 International license.

10.1128/mBio.01912-18.4 FIG S3 Effect of FtsBLQ containing FtsB mutation E56A (A) or E56K (B) on the GTase activity of PBP1b measured by continuous fluorescence assay. Download FIG S3, PDF file, 0.2 MB. Copyright © 2019 Boes et al. 2019 Boes et al. This content is distributed under the terms of the Creative Commons Attribution 4.0 International license.

### The FtsN^W83L^ mutant is less efficient in the stimulation of PBP1b activity.

The essential region of FtsN (^E^FtsN) required for the activation of sPG synthesis and cell constriction was delimited in the sequence L75 to Q93; within this region, three residues (W83, Y85, and L89) have been shown to be crucial for this activity (41). In order to establish the link between the ^E^FtsN region and PBP1b function, we have expressed and purified FtsN^Y85W^ and FtsN^W83L^ (nonpermissible mutations *in vivo*) and tested their effect on the GTase activity of PBP1b. The expression level and stability of the two mutants were similar to those of the wild type. We found that while the FtsN^Y85W^ mutant had an activating effect on PBP1b similar to that of the wild-type protein, the FtsN^W83L^ mutant was much less efficient in the stimulation of PBP1b activity (2-fold decrease in initial velocity) (Fig. 5D) and FtsN^W83L^ was unable to relieve the inhibition of PBP1b by FtsBLQ (Fig. 5E). Interestingly, analysis of the copurified PBP1b-FtsN and PBP1b-FtsN^W83L^ complexes by gel filtration showed that the W83L mutation decreases the interaction of FtsN with PBP1b. Indeed, the PBP1b/FtsN ratio was approximatively 20/80 with FtsN^W83L^ and 40/60 with FtsN^WT^ (Fig. S4). These results indicate that the ^E^FtsN (L75 to Q93) region is directly involved in the interaction and activation of PBP1b.

10.1128/mBio.01912-18.5 FIG S4 Analysis of PBP1b in complex with FtsN or FtsN_W83L_ after gel filtration. The complexes were purified by affinity purification on a nickel column followed by gel filtration. The samples were then loaded on SDS-PAGE gels and stained with Coomassie blue (A), and the amounts of proteins were analyzed by Imag Quant TL software (GE Healthcare) to determine the PBP1b/FtsN ratios (B and C). (A) Lanes: 1 and 2, FtsN control; 3, PBP1b-FtsN_W83L_; 4, PBP1b-FtsN_WT_; 5, PBP1b control. (B) Quantification result of PBP1b-FtsN_WT_ (lane 4 in panel A). (C) Quantification result of PBP1b-FtsN_W83L_ (lane 3 in panel A). Arrows 1 and 2 depict PBP1b and FtsN (or W83L) mutants, respectively. Download FIG S4, PDF file, 0.3 MB. Copyright © 2019 Boes et al. 2019 Boes et al. This content is distributed under the terms of the Creative Commons Attribution 4.0 International license.

### FtsBLQ inhibits the activity of PBP3 on a thioester substrate.

**FIG 7:**
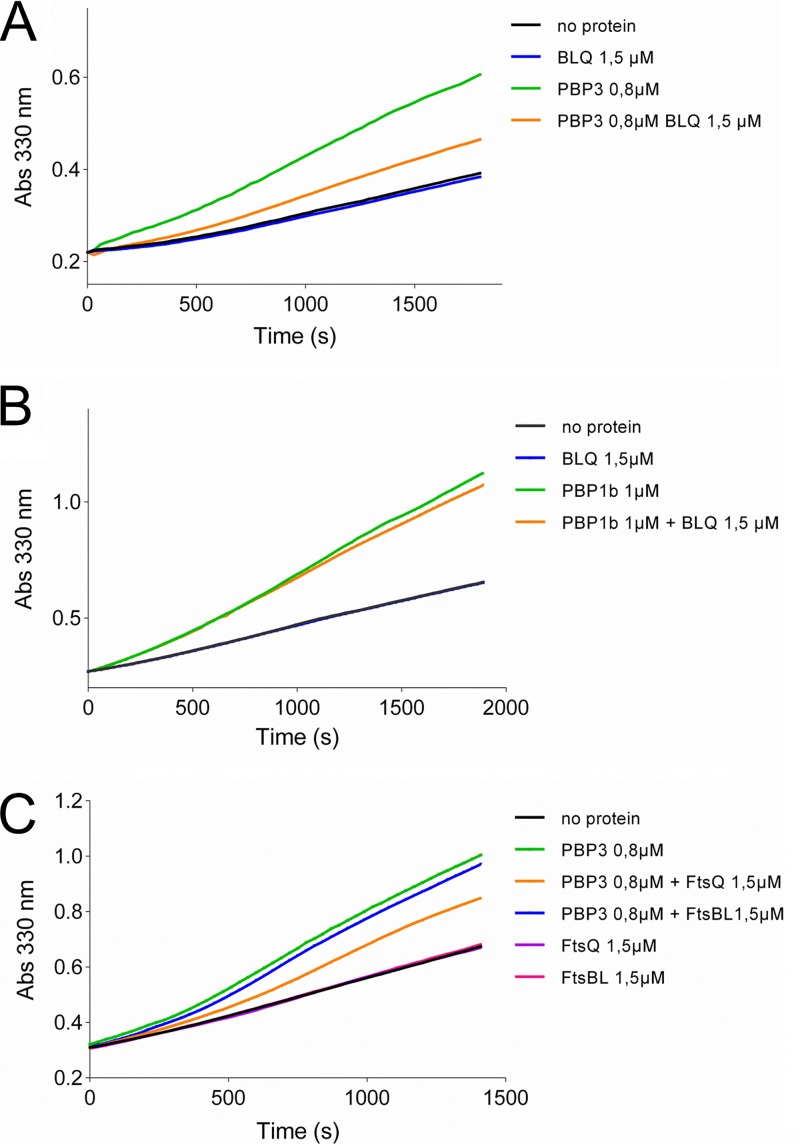
Effect of FtsBLQ and subunits on the hydrolysis of S2d by PBP3 and PBP1b TPase domain. (A) Inhibition of PBP3 activity by FtsBLQ. (B) FtsBLQ does not inhibit the hydrolysis of S2d by PBP1b TPase domain. (C) Hydrolysis of S2d by PBP3 is inhibited by FtsQ but not by FtsBL. Abs 330 nm, absorbance at 330 nm.

The purified E. coli PBP3 is able to bind penicillin and to catalyze the hydrolysis or aminolysis of simple thioester substrates, indicating that the active site is correctly folded and functional (51); in contrast, its TPase activity with the natural substrate has never been reported. In the absence of a TPase assay for PBP3 with its native substrate, we used an assay based on the thioester substrate S2d to assess the effect of FtsBLQ and mutants on its activity and that of PBP1b (52). We found that FtsBLQ inhibits the hydrolysis of S2d by PBP3 (Fig. 7A) but, in contrast, has no significant effect on the similar reaction catalyzed by the TPase domain of PBP1b (Fig. 7B). Interestingly, when FtsQ and FtsBL were added separately to the reaction, the activity of PBP3 was inhibited by FtsQ but FtsBL had only a minor effect (Fig. 7C), contrasting with the opposite effects of these subunits on the GTase activity of PBP1b. Aztreonam used as a control inhibited PBP3 as expected, and FtsBLQ or subunits had no effect on the substrate in the absence of the enzyme (Fig. 7 and Fig. S5). These results are consistent with the finding that the pneumococcal DivIB-DivIC-FtsL and DivIB (ortholog of E. coli FtsQ) were also able to decrease the activity of PBP2x (ortholog of E. coli PBP3) on S2d (53). Moreover, the observations that a region in the beta domain (residues 229 to 257) of DivIB of B. subtilis is critical for the interaction with PBP2B (ortholog of E. coli PBP3) (54) and that the A252P mutation in the corresponding region of E. coli FtsQ was found to abolish the capacity of FtsQ to recruit PBP3 strongly support our findings (55).

10.1128/mBio.01912-18.6 FIG S5 Effect of aztreonam and FtsN on the hydrolysis of S2d by PBP3. Download FIG S5, PDF file, 0.2 MB. Copyright © 2019 Boes et al. 2019 Boes et al. This content is distributed under the terms of the Creative Commons Attribution 4.0 International license.

As the FtsB mutants (FtsB^E56A^, FtsB^E56K^, or Fts^D59H^) did not affect the activity of the FtsBLQ subcomplex toward the GTase activity of PBP1b, we tested their effect on PBP3, but no change was observed (data not shown). FtsN also has no effect on the activity of PBP3 on S2d (data not shown and Fig. S5).

## DISCUSSION

How bacteria (E. coli) regulate cell division is a fundamental question. The large majority of proteins involved in cell division and that collectively form the divisome have been identified and characterized. However, the molecular mechanisms and the function of many of them remain unsolved. Initially, the FtsBLQ complex has been thought to play a scaffolding function (35, 39), but this has changed recently when suppressor mutations in FtsB or FtsL where found to completely bypass the need for FtsN, FtsA, and other divisome proteins (41, 42), suggesting an active role of FtsBLQ in the regulation of cell wall synthesis during division in coordination with FtsA and FtsN.

sPG synthesis relies on both glycan chain syntheses by the GTases (aPBP belonging to the GTase 51 family and the proteins of the SEDS family) and their cross-linking by the TPase activities of the PBPs to drive cell division. During elongation, both aPBPs and RodA have been shown to be essential for PG polymerization, as the inhibition of one of them drastically reduces cell wall synthesis (9, 56). This indicates that PBP1b and FtsW/PBP3 may also collaborate to synthesize functional sPG.

In E. coli, at least one class A PBP (PBP1a or PBP1b) is required for viability (57); although the two PBPs are exchangeable, they likely have a specific function during the cell cycle, with PBP1b exhibiting a preference for cell division (58, 59) and a clear enrichment at midcell during cell constriction, which overlap the localization of FtsN and LpoB (13, 20, 60). In the absence of PBP1b, the localization of PBP1a at midcell was shown to increase (61). In addition, it has been shown that elongasome and divisome proteins are simultaneously present at midcell for about 40% of the division cycle time (62). These observations indicate that PBP1a would take over the role of PBP1b in cell division; however, the exact mechanisms of recruitment and regulation are unknown.

Despite a compelling body of evidence linking PBP1b with cell division (10, 13, 20, 26, 27, 58–61), its exact role in this process and its regulation were overlooked. Enzymatic evidence for the positive regulation of the polymerase activity of PBP1b by the core division protein FtsN and the lipoprotein LpoB is well characterized (23, 24, 26, 63). However, while PBP1b localizes most likely with FtsW and PBP3 (20), sPG synthesis starts after FtsN accumulation to the septum, suggesting that the polymerase activity of PBP1b must also be negatively regulated.

Knowing the high activity and processivity of PBP1b *in vitro* (which are probably more impressive *in vivo*), and the restricted space at the constriction site, a small amount of PBP1b would be sufficient to drive sPG synthesis (21, 22, 64). At the division site, the copy number of PBP1b was estimated at ∼18 and that of FtsBLQ complexes at ∼20 to 66 copies per cell (60, 65). Thus, the low copy number of PBP1b at the division site is compatible with a localized regulation of its activity by the FtsBLQ complex. The cellular concentration of FtsN increases during the cell cycle by 30% to reach a maximum of ∼150 molecules per cell (60), which is ∼8 times higher than FtsBLQ and PBP1b, allowing it to efficiently compete for binding to PBP1b and trigger sPG synthesis and cell constriction.

Published data and our interaction experiments have shown multiple oligomeric interactions between FtsBLQ, FtsW/PBP3, PBP1b, and FtsN (10, 17, 26–32, 66). We refer to this group of proteins as the septum synthesis core of the divisome, composed of the synthase subcomplex (FtsW-PBP3-PBP1b) and the regulatory elements FtsBLQ and FtsN (and others such as LpoB and CpoB). Except from FtsW, the observed interactions of PBP3 with divisome partners were often weak, suggesting that they are mainly mediated by FtsW. While FtsBLQ and FtsN do not interact (or interact weakly) with each other, they directly and independently interact with higher affinity with the synthase subcomplex FtsW-PBP3-PBP1b via FtsW and PBP1b. These results are consistent with the fact that pretargeted FtsQ was not able to recruit FtsN (67), and FRET experiments showed interactions between FtsN and FtsW, FtsN, and PBP3 but lack of interaction between FtsN and FtsQ (31). On the other hand, our activity studies using different combinations of the proteins that compose the septum synthesis core of the divisome revealed that the GTase activity of PBP1b is repressed by FtsBLQ complex and that the presence of FtsN suppresses this inhibition. Moreover, we found that FtsBLQ is also able to inhibit the PBP3 activity on a thioester substrate and thus exert a double control on sPG synthesis. The inhibition of PBP1b GTase is mediated by FtsBL, which interacts with PBP1b independently from FtsQ, and that of PBP3 is mediated by FtsQ. The fact that only the FtsL^D93A^ mutant and not FtsB mutants affects the function of FtsBLQ toward PBP1b suggests that FtsL and FtsB play distinct roles as proposed elsewhere (41), that the inhibition is essentially mediated by FtsL, and that residue D93 of the CCD region plays an essential role in the specific regulation of PBP1b and could explain the physiological role of this mutant observed *in vivo* (41). This result is consistent with previous observations that FtsLs from E. coli, Bacillus subtilis, and Streptococcus pneumoniae are highly unstable in the absence of their partners FtsB/DivIC or FtsQ/DivIB, and with the high turnover of FtsL involving the membrane protease YluC in B. subtilis that was shown to regulate the level of FtsL and thereby that of the functional subcomplex (35, 68, 69). These results shed light on the molecular function of the FtsBLQ complex in the regulation of sPG synthesis and its link with FtsN, providing for the first time an enzymatic explanation for the observed *in vivo* studies and revealing PBP1b as an FtsBLQ target.

**FIG 8:**
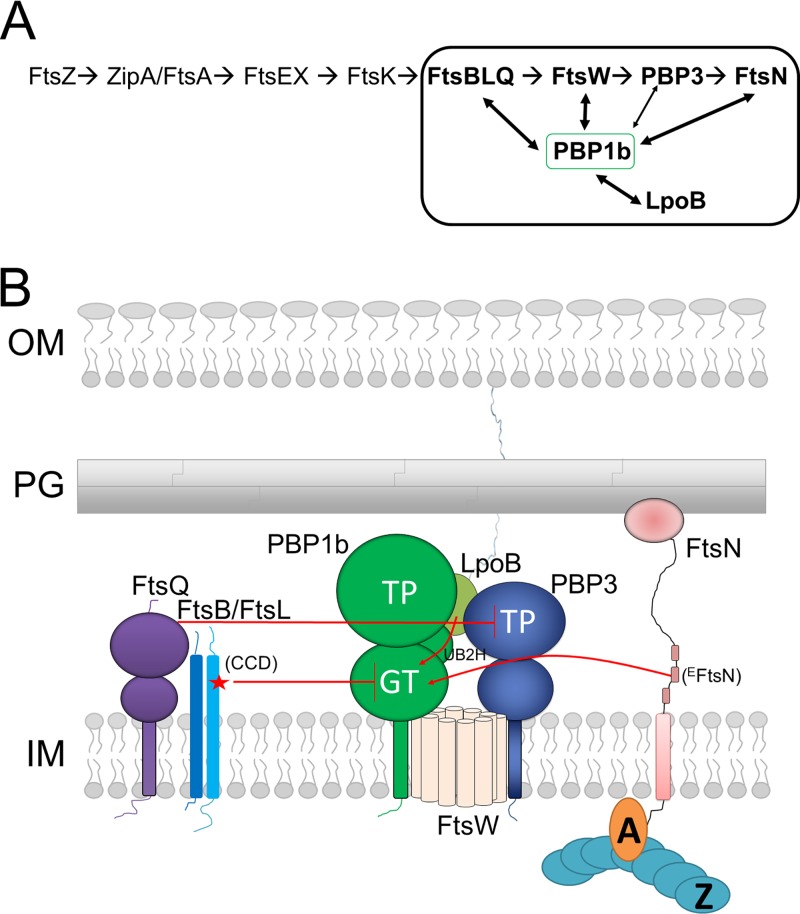
Schematic representation of the linear recruitment pathway of the divisome and a regulation model of the septal synthase subcomplex (FtsW-PBP3-PBP1b) by FtsBLQ, FtsN, and the lipoprotein LpoB. (A) Linear recruitment pathway of the divisome with the septal peptidoglycan (sPG) synthesis core shown in a frame; protein-protein interactions are shown with arrows. (B) Topology of the synthase subcomplex and the regulatory proteins (FtsBLQ, FtsN, and LpoB) in the cell envelope. FtsBLQ subcomplex inhibits the GTase activity of PBP1b (via FtsL) and the TPase domain of PBP3 (via FtsQ). Interaction of FtsN via the essential region (^E^FtsN) and of LpoB with PBP1b suppresses the inhibition by FtsBLQ, activating PBP1b and probably the whole synthase subcomplex. Interaction of FtsN with FtsA and LpoB with the outer membrane allows the coordination of sPG synthesis with cytoplasmic, inner (IM), and outer membrane (OM) events. CCD, constriction control domain of FtsL.

^CCD^FtsL (the CCD sequence of FtsL, underlined) and surrounding residues (85 to 95, **EE**NALG**D**HSRV**E**) are enriched with negatively charged residues (shown in bold). Interestingly, the ^E^FtsN sequence (residues 75 to 93, LPPKP**EE**RWRYIK**E**L**E**SRQ) is reminiscent of that of the ^CCD^FtsL with equivalent negatively charged residues, including two consecutive glutamates, suggesting that these two motifs may compete for the same binding site on PBP1b (Fig. 8). It is worth noting that the distances between the membrane surface and the GTase domain of PBP1b on the one side and the ^CCD^FtsL and ^E^FtsN motifs on the other side are similar, which would allow direct interaction between the latter motifs and the GTase domain.

Importantly, in addition to the elucidation of the role of FtsBLQ, our findings help to better define the exact function of FtsN. This protein does not simply activate PBP1b, which has a relatively high basal activity without activation. The fact that FtsN is no longer required when FtsBLQ is not functional indicates that the principal function of FtsN is to relieve the repression by FtsBLQ, probably through competition for PBP1b. The observation that the W83L mutation in the essential periplasmic region of FtsN (^E^FtsN) was less efficient in the interaction with and activation of PBP1b provides direct evidence that ^E^FtsN modulates the function of PBP1b and that the latter contributes to sPG synthesis and cell constriction. Using a thioester substrate, the activity of PBP3 was inhibited by FtsBLQ and FtsQ, but FtsN had no effect. The double inhibition of the PBP1b GTase activity and the PBP3 TPase activity by FtsBLQ would ensure a double level of control and a perfect repression of the divisome during the maturation stage. The displacement of FtsBLQ and the initiation of sPG synthesis could be allowed only by the recruitment and buildup of FtsN.

Altogether, our results and the available published data suggest that PBP1b, PBP3, and FtsW form an sPG synthase subcomplex of the divisome regulated by the antagonist activities of FtsBLQ and FtsN (Fig. 8) (in coordination with cytoplasmic events via FtsA) and LpoB (with CpoB and Tol system) coordinating sPG synthesis with outer membrane constriction (13). This organization enables a precise and balanced spatiotemporal polymerase activity of the sPG synthase subcomplex. Premature stimulation of this activity (lack of repression by FtsBLQ) or absence of derepression by FtsN results in malfunctioning of the divisome, a cell morphogenesis defect, and eventual lysis.

## MATERIALS AND METHODS

### Growth conditions.

Bacteria [E. coli C43 (DE3) or Lemo21 (DE3)] were grown in Luria-Bertani (LB) or 2× YT (for FtsN) medium supplemented with the appropriate antibiotic: ampicillin (100 µg/ml) (from MP Biomedicals), chloramphenicol (30 µg/ml) (from Sigma), or kanamycin (50 µg/ml) (from MP Biomedicals).

### Reagents.

Dansyl-lipid II and [^14^C]lipid II (0.155 µCi/nmol) were prepared as previously described (21, 70). Fluorescein-labeled ampicillin was prepared as previously described (71).

### Plasmid construction.

Plasmid constructions are described in the supplemental material (Text S1). The complete list of the plasmids used in this study is given in Table S1. All point mutations were introduced using the Q5 site-directed mutagenesis kit (New England Biolabs). The primers used in this study are shown in Table S2; they were purchased from Eurogentec (Angleur, Belgium).

10.1128/mBio.01912-18.1 TEXT S1 Supplementary materials and methods. Download Text S1, DOCX file, 0.03 MB. Copyright © 2019 Boes et al. 2019 Boes et al. This content is distributed under the terms of the Creative Commons Attribution 4.0 International license.

10.1128/mBio.01912-18.7 TABLE S1 Plasmids used in this study. Download Table S1, DOCX file, 0.02 MB. Copyright © 2019 Boes et al. 2019 Boes et al. This content is distributed under the terms of the Creative Commons Attribution 4.0 International license.

10.1128/mBio.01912-18.8 TABLE S2 Oligonucleotides used in this study. Download Table S2, DOCX file, 0.01 MB. Copyright © 2019 Boes et al. 2019 Boes et al. This content is distributed under the terms of the Creative Commons Attribution 4.0 International license.

### Expression and purification of proteins.

The following proteins were expressed and purified as previously described: E. coli PBP1b (21), LpoB (25), and FtsW-PBP3 (27).

Single proteins, the FtsBLQ complex, and the other complexes were (co)expressed in E. coli strain C43 (DE3) or Lemo21 (DE3) harboring the appropriate plasmid(s) (Table S1) and purified according to similar procedures unless mentioned elsewhere (see the supplemental material).

### Continuous fluorescence assay.

The PBP1b activity assays with dansyl-lipid II as the substrate were performed in a medium binding black 96-well microplate at 30°C (Greiner Bio One) as described previously (49, 64). The samples contained 10 µM dansyl-lipid II, 50 mM HEPES-NaOH (pH 7.5), 200 mM NaCl, 10 mM CaCl_2_, 0.085% decyl-PEG, 20% dimethyl sulfoxide (DMSO), and 1 unit of *N*-acetylmuramidase of Streptomyces globisporus (Sigma). The single proteins (FtsQ and FtsN) and complexes (FtsBLQ, FtsBL, and FtsW-PBP3) were used at indicated concentrations (0.1 to 3 µM), and LpoB was used at 200 nM. The reactions were initiated by the addition of 50 to 100 nM PBP1b and monitored by following the fluorescence decrease over 20 to 30 min at 30°C using an Infinite M200 Pro microplate reader (Tecan, Männedorf, Switzerland) with excitation at 340 nm and emission at 520 nm.

### Glycosyltransferase activity assay using TLC.

[^14^C]lipid II at 4 μM was incubated with 3 μM purified E. coli FtsBLQ complex in 50 mM HEPES-NaOH (pH 7.5), 200 mM NaCl, 10 mM CaCl_2_, 0.085% decyl-PEG, 20% DMSO, and 100 nM PBP1b for 5 min at 37°C. The reaction products were separated by thin-layer chromatography (TLC) on silica gel plates (Fluka), using 2-propanol–ammonium hydroxide (25%)–water (6:3:1, vol/vol/vol) as the mobile phase. The TLC plates were exposed to a storage phosphor screen (GE Healthcare) for 16 h, and images were revealed using a Typhoon Trio imager and Image Quant TL software (GE Healthcare).

### Hydrolysis of S2d by PBPs.

The activity of PBP3 and PBP1b (TP domains) was measured in the presence of S2d (analog of the peptide moiety) as a mimic of donor substrates as previously described (51, 52). The assay was performed in a UV-Star microplate 96-well half-area format (Greiner Bio One) at 37°C in the presence of 50 mM potassium phosphate (pH 7.0), 2.0 mM S2d, 3.2 mM 4,4′-dithiodipyridine, and 0.8 to 1 µM PBP3. The increase of absorbance at 330 nm was monitored with an Infinite M200 Pro microplate reader (Tecan, Männedorf, Switzerland). The proteins FtsQ and FtsN and the complexes FtsBL and FtsBLQ were used at indicated concentrations to test their effect on the PBP activity. The experiments were repeated at least three times with reproducible results.

### Western blotting.

After SDS-PAGE, the proteins were electrotransferred to a PVDF membrane and probed by incubation with mouse anti-His–horseradish peroxidase (HRP; Roche) or rabbit polyclonal anti-FtsB or anti-FtsL (gift from J. Luirik) or anti-FtsQ (mouse) or anti-HA (mouse) high-affinity (Roche) antibodies. Immunodetection was done using peroxidase-conjugated goat anti-rabbit or goat anti-mouse IgG secondary antibodies (Millipore). The proteins were visualized by enhanced chemiluminescence (ECL kit; GE Healthcare).

### Microscopy and image analysis.

E. coli strain C43 (DE3) harboring the appropriate plasmid(s) was grown at 37°C, in LB medium supplemented with the appropriate antibiotic to an *A*_600_ of 0.5. Then, expression was induced for 3 h by addition of 0.5 mM IPTG at 37°C. Cells were fixed, photographs were taken with a cooled AxioCam MRm (Zeiss) mounted on an Zeiss Axio Imager.Z1 microscope, and images were acquired in phase-contrast mode using the AxioVision Rel. 4.5 (Zeiss) software as previously described (72). The length of the cells was determined using ImageJ software (https://imagej.nih.gov/ij/) running under plugin ObjectJ (https://sils.fnwi.uva.nl/bcb/objectj/).
