# Supplementary material for: Regulation of the Peptidoglycan Polymerase Activity of PBP1b by Antagonist Actions of the Core Divisome Proteins FtsBLQ and FtsN
Source: mBio. 2019 Jan 8;10(1):e01912-18. doi: 10.1128/mBio.01912-18 (PMC6325244; doi:10.1128/mBio.01912-18)
Supplement: FIG S1 [file mBio.01912-18-sf001.pdf]

**Figure S1**

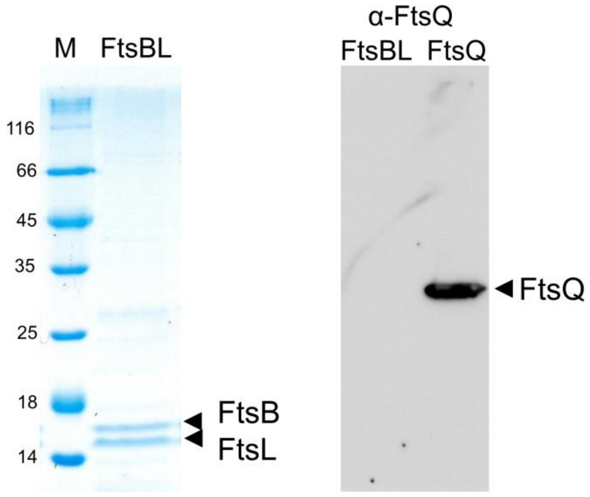


**Figure S1.** Purification of FtsBL and immunoblotting using antibodies against FtsQ. Left, Coomassie blue stained SDS-PAGE showing FtsB and FtsL bands indicated by arrows, M, molecular standards. Right (α-FtsQ), immunoblotting results confirming the absence of FtsQ in the purified FtsBL sample and FtsQ as control.
