# Supplementary material for: Regulation of the Peptidoglycan Polymerase Activity of PBP1b by Antagonist Actions of the Core Divisome Proteins FtsBLQ and FtsN
Source: mBio. 2019 Jan 8;10(1):e01912-18. doi: 10.1128/mBio.01912-18 (PMC6325244; doi:10.1128/mBio.01912-18)
Supplement: FIG S2 [file mBio.01912-18-sf002.pdf]

Figure S2

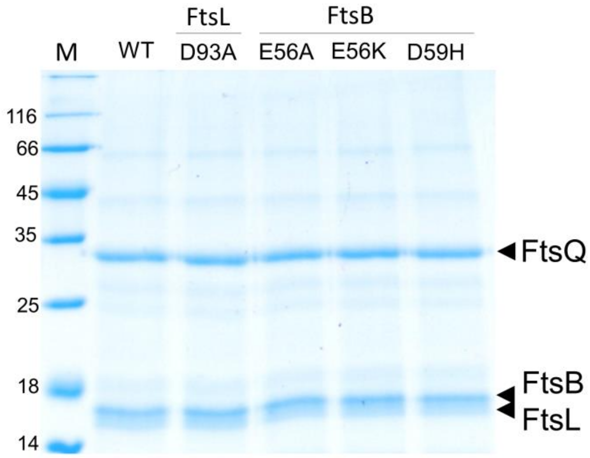

Figure S2. Coomassie blue stained SDS-PAGE showing purified FtsBLQ complex and variants containing FtsB mutations E56A, E56K or D59H or FtsL D93A. M, protein standard.
