# Supplementary material for: Regulation of the Peptidoglycan Polymerase Activity of PBP1b by Antagonist Actions of the Core Divisome Proteins FtsBLQ and FtsN
Source: mBio. 2019 Jan 8;10(1):e01912-18. doi: 10.1128/mBio.01912-18 (PMC6325244; doi:10.1128/mBio.01912-18)
Supplement: FIG S3 [file mBio.01912-18-sf003.pdf]

Figure S3

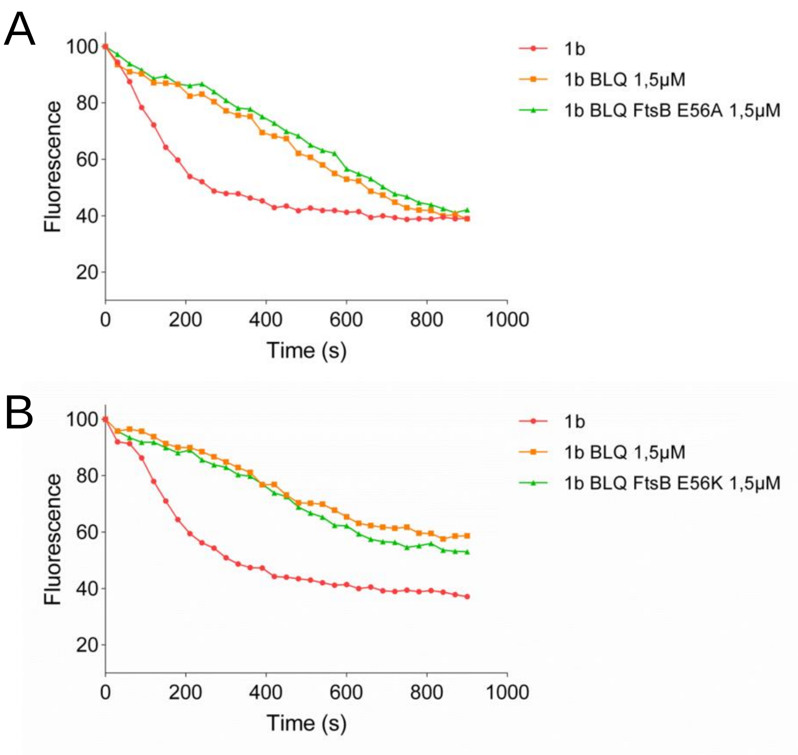

Figure S3. Effect of FtsBLQ containing FtsB mutations E56A (A) or E56K (B) on the GTase activity of PBP1b measured by continuous fluorescence assay.
