# Supplementary material for: Regulation of the Peptidoglycan Polymerase Activity of PBP1b by Antagonist Actions of the Core Divisome Proteins FtsBLQ and FtsN
Source: mBio. 2019 Jan 8;10(1):e01912-18. doi: 10.1128/mBio.01912-18 (PMC6325244; doi:10.1128/mBio.01912-18)
Supplement: FIG S4 [file mBio.01912-18-sf004.pdf]

Figure S4

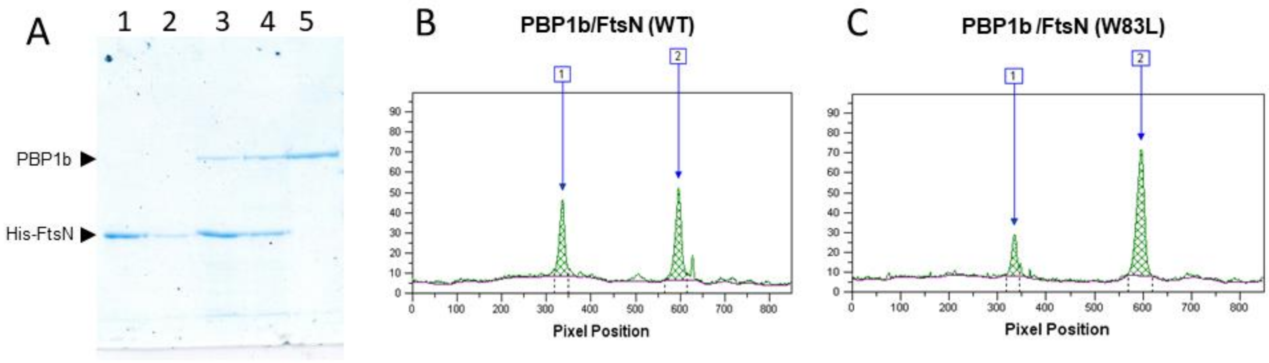

Figure S4. Analysis of PBP1b in complex with FtsN or FtsN^{W83L} after gel filtration. The complexes were purified by affinity purification on a nickel column followed by gel filtration. The sample were then loaded on SDS-PAGE, stained with Coomassie blue (A) and the amount of proteins were analyzed by Image Quant TL software (GE Healthcare) to determine the PBP1b/FtsN ratios (B and C). Panel A: 1-2 FtsN control, 3, PBP1b-FtsN^{W83L}, 4, PBP1b-FtsN^{WT}, 5, PBP1b control. Panel B: quantification result of PBP1b-FtsN^{WT} (line 4 in A). Panel C: quantification result of PBP1b-FtsN^{W83L} (line 3 in A). arrows 1 and 2 depict PBP1b and FtsN (or W83L mutants) respectively.
