# Supplementary figures and images for: Regulation of the Peptidoglycan Polymerase Activity of PBP1b by Antagonist Actions of the Core Divisome Proteins FtsBLQ and FtsN
Source: mBio. 2019 Jan 8;10(1):e01912-18. doi: 10.1128/mBio.01912-18 (PMC6325244; doi:10.1128/mBio.01912-18)

**Figure S5**

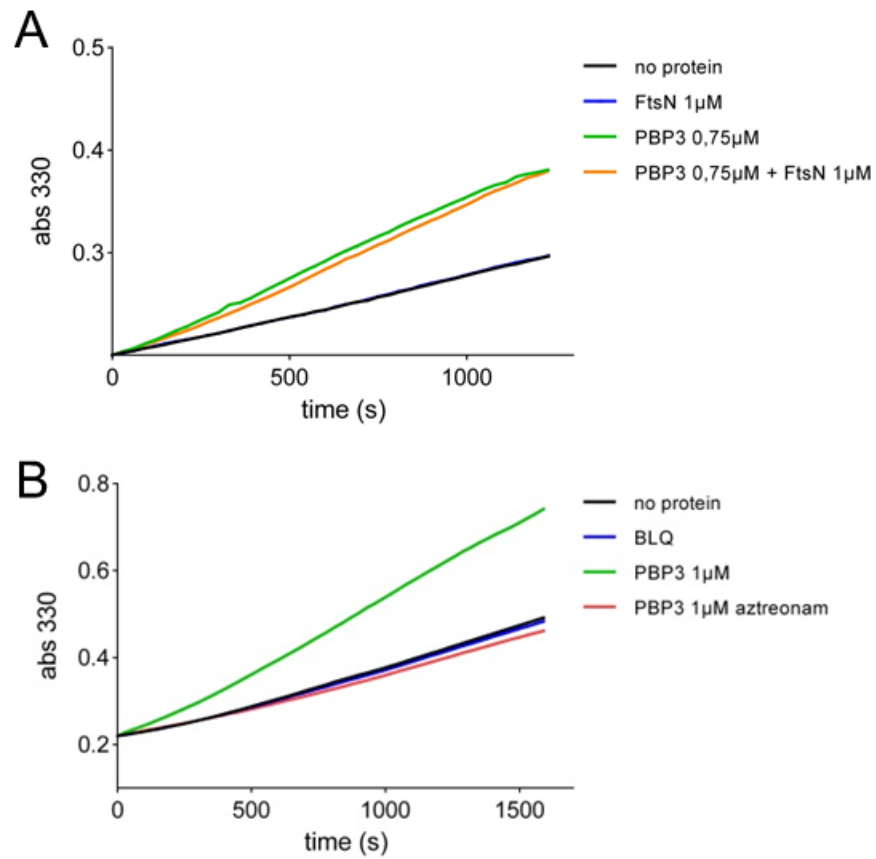

**Figure S5.** Effect of aztreonam and FtsN on the hydrolysis of S2d by PBP3

Supplement: FIG S5 [file mBio.01912-18-sf005.pdf]
