# Supplementary material for: Regulation of the Peptidoglycan Polymerase Activity of PBP1b by Antagonist Actions of the Core Divisome Proteins FtsBLQ and FtsN
Source: mBio. 2019 Jan 8;10(1):e01912-18. doi: 10.1128/mBio.01912-18 (PMC6325244; doi:10.1128/mBio.01912-18)
Supplement: TEXT S1 [file mBio.01912-18-s0001.docx]

**Supplementary Material**

**Regulation of the peptidoglycan polymerase activity of PBP1b by antagonist actions of the core divisome proteins FtsBLQ and FtsN**

**Adrien Boes^1^, Samir Olatunji^1^, Eefjan Breukink^2^ and Mohammed Terrak^1^**

^1^ InBioS-Centre d’Ingénierie des Protéines, Liège University, B6a, Quartier Agora, allée du six Août 11, 4000 Liège 1, Belgium

^2^ Membrane Biochemistry and Biophysics, Department of Chemistry, Faculty of Science,

Utrecht University, Padualaan 8, 3584 CH Utrecht, The Netherlands

**Supplementary material and methods**

**Plasmid construction**

The complete list of the plasmids used in this study is given in Table S1. The primers used in this study are shown in Table S2.

**pRSF-HisFtsBLQ and pRSF-FtsHisBL^*^Q.** The genes *ftsB, ftsL and ftsQ* were amplified by PCR using the primers BF/BR, LF/LR, and QF/QR (Table S2) respectively and ligated (via EcoRI and SalI sites) to generate the tandem sequence HindIII**-***ftsB-*(EcoRI)*-ftsL-*(SalI)*-ftsQ-*NotI. This fragment was digested by HindIII and NotI and inserted in the MCS1 of the pRSFDuet-1 vector (Novagen-EMD Millipore) between the corresponding sites. It has been shown that the expression level of the wild-type FtsL is much lower than FtsB and that its yield can be enhanced to the level of FtsB by conservative mutations at the N-terminus of the protein without affecting its function (1). In order to obtain sufficient amounts of the FtsBLQ complex, the *ftsL* gene was then mutated (S3N, R4K, and V5L) during two PCR amplification steps, using the truncated primers LmatF/LmatR and Lmat2F/L2R. The PCR fragment was digested by EcoRI and SalI and used to replace the wild-type gene giving the plasmid pRSF-HisFtsBL*^*^*Q (*ftsL^*^* contains the mutations S3N, R4K, and V5L). These constructs allow the production of His-tagged FtsB, untagged FtsL/L*^*^* and FtsQ. Only FtsL^*^ is presented in this work and will be referred to, outside this section, simply as FtsL hereafter.

**pRSF-HisFtsBL^*^Q-FtsN.** The *ftsN* gene was amplified by PCR from pDML2032 (2) using the primers NF and NR (Table S2) and inserted in the MCS2 of the pRSF-HisFtsBLQ vector between the NdeI and XhoI sites, yielding the plasmid pRSF-HisFtsBL**^*^**Q-FtsN.

**pRSF-StrepFtsBL^*^Q.** A sequence containing a Streptag II and the thrombin cleavage site flanked by the restriction sites NcoI and HindIII was obtained by oligonucleotide annealing (BLQStF and BLQStR, Table S2), and used to replace the Histag sequence between NcoI-HindIII sites of the plasmid pRSF-HisFtsBL**^*^**Q.

**pRSF-HisFtsBL^*^Q-FtsW.** *ftsW* gene was excised from the plasmid pDLM2040 using BglII and XhoI and inserted in the MSC2 of the plasmid pRSF-HisFtsBL**^*^**Q.

**pRSF-HisBL^*^Q-PBP3.** *ftsI* gene was excised from the plasmid pDLM2041 using the BglII and XhoI and inserted in the MSC2 of the plasmid pRSF-HisFtsBL**^*^**Q.

**pRSF-HisFtsBL^*^Q-PBP1b.** The *ponB* gene was amplified by PCR using the primer 1BF and 1BR (Table S2) and inserted in the MSC2 between AatII and XhoI sites of the plasmid pRSF-HisFtsBL**^*^**Q.

**pRSF-HisFtsBL^*^.** *ftsB* gene was amplified by PCR using the primers B3F and B2R and inserted in the MSC1 of the plasmid pRSF-Duet between BamHI and EcoRI sites. *ftsL***^*^** was then amplified by PCR using the primers Lmat2F and L2R (Table S2) and inserted between EcoRI and SalI.

**pRSF-HisFtsBL^*^-PBP1b.** The *ponB* gene was amplified by PCR using the primer 1BF and 1BR (Table S2) and inserted in the MSC2 of the pRSF-HisFtsBL**^*^** plasmid between AatII and XhoI sites.

**pRSF-HisFtsQ-PBP1b.** The *ponB* gene was amplified by PCR using the primer 1BF and 1BR (Table S2) and inserted in the MSC2 of the pRSF-HisFtsQ plasmid between AatII and XhoI sites.

**pRSF-HisFtsQ.** The gene *ftsQ* was amplified by PCR using the primer Q3F and Q2R and inserted in the MSC1 of the plasmid pRSF-Duet between EcoRI and NotI sites.

**pRSF-HisFtsW-FtsN.** The *ftsN* gene was amplified by PCR from pDML2032 using the primers NF and NR (Table S2) and inserted in the MCS2 of the pRSF vector between the NdeI and XhoI sites. Then *ftsW* gene was excised from the plasmid pDML2041 using BamHI/HindIII and inserted in the MCS1.

**pDuet-HisFtsN-PBP1b**. *fts*N was amplified by PCR from pDML2032 using N2F and N2R and inserted in the MCS2 of the pDuet vector between BamHI/ HindIII. Then the *ponB* gene was amplified by PCR using the primer 1BF and 1BR (Table S2) and inserted in the MSC2 between AatII and XhoI sites.

**pRSF-HisFtsBL^*^Q-FtsW_HA_.** *fts*W_HA_ gene was excised from the plasmid pDML2043 by BglII/XhoI and insert in pRSF-HisFtsBL**^*^**Q plasmid in the MCS2.

**Expression and purification of proteins**

The following proteins were expressed and purified as previously described: *E. coli* PBP1b (Terrak et al 1999), LpoB (3) and FtsW-PBP3 (4).

Single proteins, the FtsBLQ complex and the other complexes were (co-)expressed in *E. coli* strain C43 (DE3) or Lemo21 (DE3) harboring the appropriate plasmid(s) (Table S1) and purified following similar procedures unless mentioned elsewhere. Bacteria were grown at 37°C, in LB medium supplemented with the appropriate antibiotic to an A_600nm_ of 0.8. Then expression was induced for 3.5 hours by addition of 0.5 mM isopropyl β-D-1-thiogalactopyranoside (IPTG) at 30 °C and 100 µM rhamnose when Lemo21 (DE3) was used as host strain. Cells were collected by centrifugation at 4000 × *g* for 20 minutes at 4°C and resuspended in a buffer containing 50 mM Tris–HCl, pH 7.5, 50 mM NaCl, and EDTA-free protease inhibitor Cocktail (Roche). The bacterial cells were lysed by three passages through a cell homogeneizer (Emulsiflex C3 Avestin®). After centrifugation at 4000 × *g* for 15 minutes at 4°C, the supernatant was recovered and was spun down at 150,000 × g for 1 hour at 4°C and then the membranes were solubilized in 50 mM HEPES-NaOH pH 7.5, 500 mM NaCl, 10% (v/v) glycerol, 40 mM n-dodecyl-β-D-maltopyranoside (DDM; Inalco®) and complete EDTA-free protease inhibitors. The mixture was incubated for 1 hour at room temperature under gentle agitation followed by centrifugation at 150,000 × g for 1 hour at 4°C, the supernatant containing the solubilized membrane proteins or complexes were purified by affinity chromatography. The samples were loaded onto a HisTrap (GE HealthCare) or a Streptactin (IBA Lifescience) column conditioned in buffer A (50 mM HEPES-NaOH, pH 7.5, 300 mM NaCl, 10% (v/v) glycerol and 1 mM DDM). For HisTrap, buffer A contained in addition 50 mM imidazole and the protein or protein complexes were eluted by an imidazole gradient (0.05-1 M). For the Streptactin, the proteins were eluted with 2.5 mM desthiobiotin in buffer A. After SDS-PAGE analysis, pure fractions were pooled and desalted on a G25 Sephadex column and stored at -20°C. The concentrations of the proteins or complexes were determined by measuring the absorbance at 280 nm and with the predicted extinction coefficient (ProtParam).

The samples containing PBPs were labelled with 10 µM fluorescent ampicillin for 30 minutes at 37°C, before the analysis by SDS-PAGE. The labelled PBPs were then visualized using a Typhoon Trio+ (GE Healthcare). Then the gels were stained with Coomassie blue.

**Expression and purification of FtsN.** *E. coli* C43 (DE3) cells containing the plasmid pDML2032 were grown in 2xYT medium at 37°C and induction performed at A_600nm_ of 0.6 with 0.5 mM IPTG for 3 hours. The cells were suspended in 25mM Tris-HCl pH 7,5, 25 mM NaCl and disrupted using a cell homogeneizer (Emulsiflex C3 Avestin®). After centrifugation at 4000 × *g* for 15 minutes at 4°C, the supernatant was recovered and was spun down at 150,000 × g for 1 hour at 4°C. The membranes were solubilized in 50 mM Tris-HCl pH 7,5, 1M NaCl, 20 % (v/v) glycerol and 40 mM (w/v) DDM for 1 hour at room temperature followed by centrifugation at 150,000 × g for 1 hour at 4°C. The supernatant was mixed with an equivalent volume of (66 % saturation) ammonium sulfate and incubated overnight at 4°C and the protein was recovered by centrifugation at 13,500 × g for 25 min at 4°C and suspended in 25 mM Tris-HCl pH 7,5, 0,5 M NaCl, 10 % (v/v) glycerol, 4 mM DDM. FtsN was then purified on the HisTrap column in buffer 25 mM Tris-HCl pH 7,5, 0,5 M NaCl, 10 % glycerol, 4 mM (w/v) DDM. Protein fractions were pooled and desalted on a G25 Sephadex column and concentrated using an Amicon apparatus (EMD Millipore) with a 10 kDa cutoff followed by gel filtration on a Superdex 200 Increase 10/300 GL column.

**Gel filtration and quantification of PBP1b in complex with FtsN and FtsN^W83L^ mutant.**

Gel filtration experiments were performed after affinity purification of the complexes using a Superdex 200 Increase 10/300 GL column in buffer 25 mM Tris-HCl pH 7,5, 500 mM NaCl, 10% (v/v) glycerol and 4 mM (w/v) DDM. The Quantification of PBP1b and FtsN bands was performed using Image Quant TL software (GE Healthcare).

**Supplementary references**

1. van den Berg van Saparoea HB, Glas M, Vernooij IGWH, Bitter W, den Blaauwen T, Luirink J. 2013. Fine-mapping the contact sites of the Escherichia coli cell division proteins FtsB and FtsL on the FtsQ protein. J Biol Chem 288:24340–50.

2. Müller P, Ewers C, Bertsche U, Anstett M, Kallis T, Breukink E, Fraipont C, Terrak M, Nguyen-Distèche M, Vollmer W. 2007. The Essential Cell Division Protein FtsN Interacts with the Murein (Peptidoglycan) Synthase PBP1B in Escherichia coli. J Biol Chem 282:36394–36402.

3. Egan AJF, Jean NL, Koumoutsi A, Bougault CM, Biboy J, Sassine J, Solovyova AS, Breukink E, Typas A, Vollmer W, Simorre J-P. 2014. Outer-membrane lipoprotein LpoB spans the periplasm to stimulate the peptidoglycan synthase PBP1B. Proc Natl Acad Sci U S A 111:8197–202.

4. Leclercq S, Derouaux A, Olatunji S, Fraipont C, Egan AJF, Vollmer W, Breukink E, Terrak M. 2017. Interplay between Penicillin-binding proteins and SEDS proteins promotes bacterial cell wall synthesis. Sci Rep 7.
