# Supplementary material for: Regulation of the Peptidoglycan Polymerase Activity of PBP1b by Antagonist Actions of the Core Divisome Proteins FtsBLQ and FtsN
Source: mBio. 2019 Jan 8;10(1):e01912-18. doi: 10.1128/mBio.01912-18 (PMC6325244; doi:10.1128/mBio.01912-18)
Supplement: TABLE S1 [file mBio.01912-18-st001.docx]

**Table S1. Plasmids used in this study**

| **Plasmid** | **Description** | **Reference** |
| --- | --- | --- |
| pDML924 | His-PBP1bγ | (1) |
| pDML2032 | FtsN-His | (1) |
| pDML2040 | His-PBP3 / FtsW | (5) |
| pDLM2041 | His-FtsW / PBP3 | (7) |
| pCIP1000 | His-FtsW / PBP1b | (7) |
| pDML2043 | His-PBP3 / FtsW_HA_ (HA epitope in 7/8 loop) | (7) |
| pRSF-His*fts*BLQ | His-FtsB/FtsL/FtsQ | This study |
| pRSF-His*fts*BL^*^Q | His-FtsB/FtsL*/FtsQ (* S3N, R4K and V5L in FtsL) | This study |
| pRSF-His*fts*BL^*^Q-*fts*N | His-FtsB / FtsL^*^/ FtsQ / FtsN | This study |
| pRSF-His*fts*BL^*^Q-*fts*W | His-FtsB / FtsL^*^/ FtsQ / FtsW | This study |
| pRSF-His*fts*BL^*^Q-*ftsI* | His-FtsB / FtsL^*^/ FtsQ / PBP3 | This study |
| pRSF-His*fts*BL^*^Q-*pon*B | His-FtsB / FtsL^*^/ FtsQ / PBP1b | This study |
| pRSF-Strep*fts*BL^*^Q | Strep-FtsB / FtsL^*^/FtsQ | This study |
| pRSF-HisW-FtsN | His-FtsW / FtsN | This study |
| pDuet-His*fts*N-*pon*B | His-FtsN / PBP1b | This study |
| pRSF-His*fts*BL^*^ | His-FtsB / FtsL^*^ | This study |
| pRSF-His*fts*Q | His-FtsQ | This study |
| pRSF-His*fts*BL^*^-*pon*B | His-FtsB / FtsL^*^ / PBP1b | This study |
| pRSF-HisFtsQ-*pon*B | His-FtsQ / PBP1b | This study |
| pRSF-His*fts*BL^*^Q-*fts*W_HA_ | His-FtsB / FtsL^*^/FtsQ / FtsW_HA_ | This study |
| pRSF-His*fts*BL^*^Q (FtsB E56A) | His-FtsB^E56A^ / FtsL^*^/ FtsQ | This study |
| pRSF-His*fts*BL^*^Q (FtsB E56K) | His-FtsB^E56K^ / FtsL^*^/ FtsQ | This study |
| pRSF-His*fts*BL^*^Q (FtsB D59H) | His-FtsB^D59H^ / FtsL^*^/ FtsQ | This study |
| pRSF-His*fts*BL^*^Q (FtsL^*^ D93A) | His-FtsB / FtsL^*^/ FtsQ (FtsL D93A) | This study |
| pRSF-His*fts*BL^*^Q-*pon*B (FtsL^*^ D93A) | His-FtsB / FtsL^*D93A^ / FtsQ / PBP1b | This study |
| pET22b-*fts*N-His (W83L) | FtsN^W83L^ -His | This study |
| pET22b-*fts*N-His (Y85W) | FtsN^Y85W^ -His | This study |
