# Supplementary material for: Regulation of the Peptidoglycan Polymerase Activity of PBP1b by Antagonist Actions of the Core Divisome Proteins FtsBLQ and FtsN
Source: mBio. 2019 Jan 8;10(1):e01912-18. doi: 10.1128/mBio.01912-18 (PMC6325244; doi:10.1128/mBio.01912-18)
Supplement: TABLE S2 [file mBio.01912-18-st002.docx]

**Table S2. Oligonucleotides used in this study**

| **Primer** | **Sequence (5’-3’)** |
| --- | --- |
| QF | ATGTCGCAGGCTGCTCTGAACAC |
| QR | TCATTGTTGTTCTGCCTGTGCCTG |
| Q2F | TTGAGTCGACAATAATTTTGTTTAACTTTAAGAAGGAGATATACATATGTCGCAGGCTGCTCT |
| Q2R | CAGAGCGGCCGCTCATTGTTGTTCTGCCTGTGCCTG |
| LF | ATGATCAGCAGAGTGACAGAAGCTC |
| LR | TTATTTTTGCACTACGATATTTTCTTGTGAC |
| L2F | TTGAGAATTCAATAATTTTGTTTAACTTTAAGAAGGAGATATACATATGATCAGCAGAGT |
| L2R | CAGAGTCGACTTATTTTTGCACTACGATATTTTCTTGTGA |
| BF | ATGGGTAAACTAACGCTGCTGTGGC |
| BR | TTATCGATTGTTTTGCCCCGCAGAC |
| B2F | CAGAAAGCTTACGCTGCTGTTGCTGGCTATTCTG |
| B2R | CAGAGAATTCTTATCGATTGTTTTGCCCCGCAGAC |
| LmatF | ATGATTAATAAATTAACAGAAGCTCTAAGCAAAGTTAAAGGATC |
| LmatR | TTATTTTTGCACTACGATATTTTCTTGTGACG |
| Lmat2F | TTGAGAATTCAATAATTTTGTTTAACTTTAAGAAGGAGATATACATATGATTAATAAATTAAC |
| BLQStF | CATGGCAAGCTGGAGCCACCCGCAGTTCGAAAAGGGTGCACTGGTGCCACGCGG |
| BLQStR | AGCTTACCAGCGGAACTACCGCGTGGCACCAGTGCACCCTTTTCGAACTGCGGG |
| NF | TATACATATGGCACAACGAGATTATGTACG |
| NR | CAGACTCGAGTCAACCCCCGGCGGCGAGC |
| 1BF | TATAGACGTCATGCCGCGCAAAGGTAAGGGCAAAG |
| 1BR | CAGACTCGAGTTAATTACTACCAAACATATCCTTGATCCAAC |
| B3F | TATAGGATCCGGGTAAACTAACGCTGCTGTTGCTGG |
| Q3F | TATAGAATTCGTCGCAGGCTGCTCTGAACACG |
| B1E56A | ACTTTTTGCCGCGATTGACGATCTC |
| B2E56A | TGATCGTTTCGCGCTTTAAG |
| B1 E56K | ACTTTTTGCCAAAATTGACGATCTC |
| B2 E56K | TGATCGTTTCGCGCTTTAAG |
| B1D59H | CGAAATTGACCATCTCAATGGCG |
| B2D59H | GCAAAAAGTTGATCGTTTC |
| L1D93A | TGCGCTCGGCGCGCATAGCCGGG |
| L2D93A | TTCTCTTCAAGGATCAGGTTGCGC |
| N1Y85W | ACGCTGGCGCGCGATTAAAGAGCTGG |
| N2Y85W | TCTTCTGGTTTTGGTGGTAG |
| N1 W83L | AGAAGAACGCCTGCGCTACATTAAAG |
| N2 W83L | GGTTTTGGTGGTAGTCCG |
